# Mechanical Deformation and Electronic Structure of a Blue Copper Azurin in a Solid-State Junction

**DOI:** 10.3390/biom9090506

**Published:** 2019-09-19

**Authors:** Carlos Romero-Muñiz, María Ortega, J. G. Vilhena, Ismael Diéz-Pérez, Juan Carlos Cuevas, Rubén Pérez, Linda A. Zotti

**Affiliations:** 1 Departamento de Física Teórica de la Materia Condensada, Universidad Autónoma de Madrid, E-28049 Madrid, Spain; 2 Department of Physics, University of Basel, Klingelbergstrasse 82, CH-4056 Basel, Switzerland; 3 Department of Chemistry, Faculty of Natural & Mathematical Sciences, King’s College London, Britannia House, 7 Trinity Street, London SE1 1DB, UK; 4 Condensed Matter Physics Center (IFIMAC), Universidad Autónoma de Madrid, E-28049 Madrid, Spain

**Keywords:** azurin, solid-state junction, biomolecular electronics, electronic transport, density functional theory, molecular dynamics

## Abstract

Protein-based electronics is an emerging field which has attracted considerable attention over the past decade. Here, we present a theoretical study of the formation and electronic structure of a metal-protein-metal junction based on the blue-copper azurin from pseudomonas aeruginosa. We focus on the case in which the protein is adsorbed on a gold surface and is contacted, at the opposite side, to an STM (Scanning Tunneling Microscopy) tip by spontaneous attachment. This has been simulated through a combination of molecular dynamics and density functional theory. We find that the attachment to the tip induces structural changes in the protein which, however, do not affect the overall electronic properties of the protein. Indeed, only changes in certain residues are observed, whereas the electronic structure of the Cu-centered complex remains unaltered, as does the total density of states of the whole protein.

## 1. Introduction

In the emerging field of biomolecular electronics, proteins represent one of the most promising candidates to be incorporated as active elements in solid-state devices. For instance, their capability of catalyzing a large number of reactions together with their intrinsic possibilities in terms of chemical recognition and selectivity make them ideal to be employed as sensors [1]. In addition, they can undergo electromechanical and optoelectronic processes; for this reason, possible ways of incorporating them in field-effect transistors, electrical noses and solar cells have recently been pursued [2]. Flexible implants based on proteins have also been suggested as they would offer the advantage of biocompatibility. Furthermore, the electron-transport properties of these systems are remarkable. Current flow through proteins has been found to be much more efficient than expected [3,4], although its exact mechanism is still under debate. An example is given by azurins, which are a class of redox metalloproteins involved in the denitrification processes, whose function is related to electron shuttling between cytochrome enzymes [5]. For this reason, their electronic properties have been studied in depth [6,7,8,9,10,11,12,13]. The mechanism for electron transport through these systems within solid-state junctions remains, however, unclear. For instance, both sequential tunneling and fully-coherent tunneling have been proposed as plausible transport scenarios in the case of blue-copper azurins [14,15,16]. Based on recent *ab initio* results for this protein in its isolated state, it cannot be ruled out *a priori* that moieties other than the Cu ion do not play a role in the electron transport [11]. These interesting results highlight the need for further theoretical investigation of a protein-based electronic device, i.e., with a Cu azurin, at the atomic-scale.

**Figure 1:**
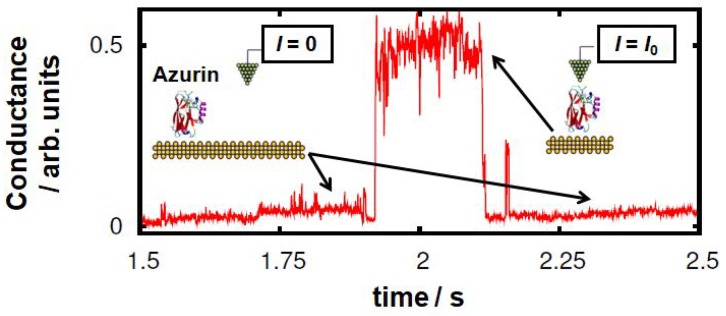
Representative “blink” identified in the experimental transients of the conductance relative to the current flowing between the two electrodes at a constant distance (2.5–3 nm) and V${}_{bias}=20$ mV (from 1.921 s to 2.113 s in this example). This kind of event is observed when the azurin protein spans the gap between the STM tip and the Au substrate electrodes. When the protein detaches itself from one of the electrodes, the conductance drops down to the initial set point level (50 pA).

A first step towards this end is the study of the conformational changes the azurin protein can undergo when placed between two metal electrodes and how they affect its electronic structure. The conductance of this protein has so far been measured by various techniques [14,15,17,18,19], including STM (Scanning Tunneling Microscopy) which has been exploited in both the break-junction and blinking modes [14]. The latter is particularly suitable for measuring proteins, as it allows evaluation of single-protein conductance whilst minimizing disturbance of the protein’s secondary structure. In this technique, the tip is brought into close proximity with an individual protein, avoiding mechanical pulling, and kept at a fixed distance from the surface. The transient current is then monitored. Occasionally, contact is created between the STM tip and the protein due to thermal fluctuations of the molecule which induce its spontaneous attachment or detachment to/from the tip (this kind of event will be henceforth called “blinking”). When this happens, jumps in the current signal are detected (see representative trace in Figure 1). This technique was initially applied to small organic molecules [20,21,22,23]. Later, the same technique was improved by adding a pulling step subsequent to the blinking event in order to corroborate the presence of a molecule in the junction. This has made it possible to measure the conductance of more complex organic molecules [24] or metal-organic complexes like porphyrins [25]. These measurements have proven highly sensitive to the specific target molecule showing a clear dependence on its size and nature and even to subtle conformational changes or the orientation and the tilting angle of the molecular junction. In addition, it has been demonstrated that this technique can be used to trigger and monitor on-surface chemical reactions [25]. Applying it to small-size biomolecules such as azurins [14], ensures a minimal impact on the general folding structure of the protein as compared with the STM break-junction technique: there, the tip is brought into physical contact with the protein-support surface followed by a fast pulling of the protein junction, causing possible protein deformation. It is important to note, however, that the two techniques have shown quite consistent results on the blue-copper azurin [14], indicating the high mechanical stability of this globular system. From a theoretical perspective, numerous theoretical studies have been carried out to investigate the relationship between the geometric structures of small molecular junctions and the measured conductance [26,27]; moreover some of the long-standing controversial issues have by now been clarified [28]. However, very little has been done in the case of proteins (mainly because their larger size makes the calculations very computationally demanding) and not much is known about the actual geometrical conformation of these systems in the junctions at the atomic scale. Quite often, the insertion of groups which are known to bind well to the metal electrodes, such as cysteine, ensures a certain directionality of the junction. However, given the level of complexity in the structure of a protein, many factors remain unclear. This includes, for instance, the exact mechanisms concerning the interaction between the target protein and the tip in a blinking event.

**Figure 2:**
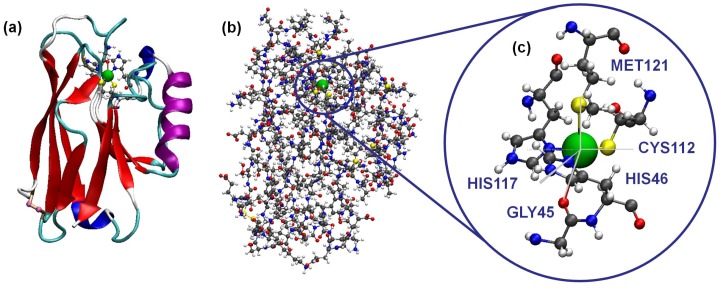
Structure of the blue copper azurin of *Pseudomonas aeruginosa* used in this work. (**a**) Ribbon representation of the tertiary structure of the protein, showing the amino acids of the first coordination sphere of the Cu center. (**b**) Ball-and-stick model of the whole protein. (**c**) Detailed view of the Cu complex of the protein displaying the five residues of the first coordination sphere with its typical geometry of distorted trigonal bipyramid.

This work tries to shed new light on this aspect. We used a combination of molecular dynamics (MD) simulations and Density Functional Theory (DFT) calculations to track the blinking event of a single blue copper azurin confined between a metallic substrate and a gold tip. More specifically, we focused on the azurin from *Pseudomonas aeruginosa*. In Figure 2, we show both ribbon and ball-and-stick representations of this protein, together with a zoomed-in view of the Cu complex. The MD simulations allow us to reproduce the blinking dynamics capturing the approaching movement of the biomolecule to the tip, which takes place through some key charged residues that promote the molecule-tip interaction. Secondly, we analyzed how the electronic properties of the protein are affected by the contact between the azurin and the tip. We see that the electronic levels associated with the Cu complex remain unchanged during the blinking event whereas significant changes are observed in some residues which become closer to the tip/substrate.

## 2. Methods

### 2.1. Molecular Dynamics

**MD simulation details.** All the simulations were performed using the AMBER14 software suite [29] with NVIDIA GPU acceleration [30,31,32]. The ff14SB force field [33] was used to describe all standard amino acids present in the azurin. The inter-atomic potentials of the copper atom and its corresponding 5 ligands were described using a quantum mechanically derived force field [34]. This force-field includes both bonded and non-bonded terms between the Cu${}^{2+}$ atom and its 5 ligands and has been widely used to model the blue-copper azurin protein [14,35,36,37,38]. In particular recent experiments [36] have shown how early stages of mechanical unfolding of this protein are well described by this force-field. The X-ray crystallographic structure of azurin was obtained from the protein data bank [39] with the PDB code 4AZU [40]. Protons were added to the protein structure according to the calculated ionization states [41] of its titratable groups at a pH of 4.5, which corresponds to the one used in the experiments of ref. [14]. The net charge of the resulting structure was zero. In all our simulations the system is fully embedded in a water medium. The water is described using explicit TIP3P model [42]. We used periodic boundary conditions with a rectangular box (∼121×121×99 Å) and Particle Mesh Ewald to account for long-range electrostatic interactions. Van der Waals contacts were truncated at the real space cutoff of 10 Å. The SHAKE algorithm was used to constrain bonds containing hydrogen, thus allowing us to use an integration step of 2 fs. Coordinates were saved every 1000 steps.

The gold atoms were described using CHARMM-METAL [43,44], which is thermodynamically consistent with the force field used to describe the protein and it has been successfully employed to study similar inorganic-bio-molecular interfaces [44]. We considered one Au(111) surface composed of three atomic layer-thick slab, where the positions of the atoms in the lowest layer are fixed during the MD runs using an harmonic restrain of 5 kcal/mol. The dimensions of the considered surface were $8\times 8\;{\mathrm{nm}}^{2}$ along the xy plane. The gold tip used to perform the indentation has a radius of 2 nm and the Au(111) planes are perpendicular to the indentation direction.

**MD simulation protocol.** The simulation protocol consisted of a two stage process: (a) Free adsorption of the azurin to Au(111); and (b) indentation of the as adsorbed azurin with an Au tip. In both cases: *(i)* The energy of the structures was minimized to avoid steric clashes using a combination of steepest descent and conjugate gradient methods; *(ii)* subsequently, the system pressure and the temperature were stabilized at ambient conditions (T=300 K and P=1 atm) using a 2 ns long NPT simulation to obtain a well-characterized water distribution under those conditions (note that all simulations are performed in liquid medium); and, finally, *(iii)* the production runs were performed in the NVT ensemble, considering that the system pressure was already stabilized in the previous stage and that the computational cost associated to NVT simulations is lower with respect to NPT. Concerning the free adsorption stage, the protein is initially positioned at 1 nm from the surface and is then allowed to freely adsorb to Au(111) in a 150 ns long NVT MD simulation. As for the indentation stage, we start from the equilibrium configuration obtained in (a) and we introduce the Au tip (with the corresponding solvating water molecules) at 3.8 nm from the surface, thus avoiding any initial interaction between tip/protein. Then we approach the tip to the surface at a velocity of 0.2 m/s until the distance between the tip and the upmost atom of the azurin is 1.2 nm, i.e., not in contact. Then we keep the position of the tip fixed (by restraining the topmost layer with an harmonic restraint of 5 kcal/mol) for the following 500 ns of simulations (performed in the NVT ensemble).

### 2.2. Density Functional Theory

Density functional theory (DFT) calculations were carried out on the azurin protein placed between a Au(111) substrate and a metallic tip of gold. The input geometries were extracted from the MD simulations described above. We used the OpenMX open source package, which is an efficient DFT code based on highly optimized numerical pseudoatomic orbitals (PAOs) [45,46]. We followed a similar approach to that we used in our previous work on gas-phase azurins [11] and cytochrome C [47]. The initial geometries for the DFT calculations were obtained from selected frames of the previous MD simulations, including the whole protein (1929 atoms) and the parts of the substrate and the tip closest to the protein. The water molecules surrounding the junction were removed. The gold substrate was modelled as a rectangular three-layer slab with a total dimension of around 54.6×42.3 Å, while the tip was constructed by cutting a (111) surface to produce a hemisphere shape. The substrate and the tip employed in these calculations consist of 969 and 252 atoms, respectively.

We used the Perdew-Burke-Ernzerhof (PBE) exchange and correlation functional [48] and norm-conserving pseudopotentials [49]. Single-ζ basis sets were used for all species involved in the calculations (including the gold tip and substrate) except for the Cu center. In this latter case, a double-ζ basis set was employed. The cutoff radii of the PAOs were carefully selected, namely 8 Bohr (Cu), 7 Bohr (S) and 5 Bohr (C, N, O and H). For the Au atoms belonging to the tip and the substrate the cutoff radii was chosen as 7 Bohr for atoms in the inner regions of the tip and the substrate and 9 Bohr for the outer region of the tip and the uppermost layer of the substrate. The electronic self-consistency was achieved by a Pulay mixing scheme based on the residual minimization method in the direct inversion iterative subspace (RMM-DIIS) [50] with a Kerker metric [51], using an energy cutoff of $10^{-8}$ Ha as convergence criterion. It is worth noting that we left the system to explore the phase space (∼500 iterations) before apply the Pulay mixing scheme. This choice prevents spurious effects arising from numerical instabilities due to the large size of the matrices involved in the calculation. The electronic temperature was set to 1200 K to facilitate convergence, which is still appropriate to provide fully consistent results. The subsequent evaluation of the projected density of states has been carried out separately using a gaussian smearing of 0.01 eV.

## 3. Results and Discussion

**Figure 3:**
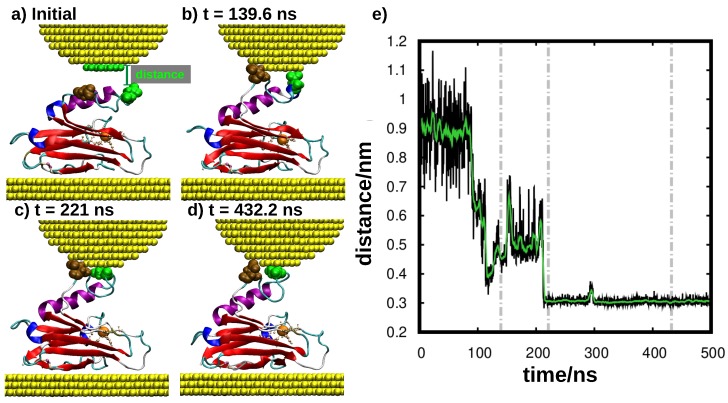
Dynamics of blinking tip-protein-surface contacts. (**a**–**d**) molecular dynamics (MD) simulation snapshots at representative tip-protein distances. The selected frames are highlighted via a gray dashed lined in panel (**e**). The surface/tip Au atoms are represented as yellow spheres, and the protein is represented using its secondary structure (β-sheets in red, α-helix in purple, turns in cyan and random coils in gray). Note that the water molecules were not represented for clarity purposes. The two main anchoring amino acids in the protein-tip contact, i.e., the aspartic acids 76 and 69, are marked in brown and green color, respectively. (**e**) Time evolution of the distance between the tip and the topmost azurin atom (residue 69) in the MD simulations. The distance is defined as the difference between the *z* coordinate of the bottommost tip layer geometric center and the geometric center of the residue 69. In black, we represent the raw data, and in green, its running average over 6 ns time frames.

In this work, we analyzed the mechanism of contacting an azurin protein adsorbed on a gold surface with an STM tip using the blinking technique. We focused on the case in which the tip is brought into proximity to the protein from above. Other kinds of scenarios could be possible, in which, for instance, the tip approaches the protein sideways and which were not considered in the present work. In order to obtain an atomistic understanding of the dynamics of single-protein junctions in the blinking modality [14], we performed molecular dynamics (MD) simulations of a tip-azurin-surface junction as detailed in the methods section. The position of the tip with respect to the surface is held at a fixed distance for the 500 ns of simulations. During the first 90 ns of simulation, the topmost atom in the azurin is 1.2 nm from the tip (see Figure 3a,e). Despite being anchored through its β-barrel laying flat over the surface, the azurin exhibits a pronounced mobility promoted by the soft/flexible random coil connecting the β-barrel to the α-helix giving rise to up/down motion of the latter. Interestingly, this enhanced flexibility allows the azurin to contact the tip positioned above just after 90 ns of simulation (see Figure 3b,e). The contact is initially established by the residue 76, which is an aspartic acid located in a flexible random-coil region connecting the α-helix to the β-barrel (see Figure 3a). Using this as an anchoring point, the tip-protein contact evolves with more amino acids becoming attached. In particular, at 139 ns, we observed that a second amino acid (residue 69) also establishes a net interaction with the tip. This second amino acid is also an aspartic acid located at the end of the α-helix (see Figure 3b). The adhesion of these two amino acids to the tip, i.e., residues 76 and 69, completely stabilizes the structure of the α-helix about 1 nm away from the rest of the azurin atoms (see Figure 3b), leading to a general azurin structure where the α-helix arm is quite separated from the β-barrel. This “opened ” azurin structure is maintained until the end of the simulation (500 ns), even when the amino acid 69 slides over the tip/surface at t∼210 ns (see Figure 3c,e) causing the amino acid 76 to separate slightly from the tip at t∼300 ns (see Figure 3d). Note that these simulations only relate to the initial tip-protein attachment and its stabilization, thus not describing the current drop observed in Figure 1 at the end of the blinking event. This is probably due to the fact that certain effects (such as the tip drift) which cause the tip to detach from the protein are not included in our simulations. Moreover, the MD time frame is not long enough as to reach the point in which the junction breakdown takes place in the experiments.

First-principles calculations were used to track the evolution of the electronic properties of the protein during the blinking event. We studied the four selected geometries extracted from the MD simulations as inputs for the DFT calculations. The first geometry, labeled as t=0 (or initial) represents the starting point when the protein is adsorbed on the substrate but still relatively far from the tip. The other three configurations labeled with the corresponding time in ns of the MD simulation correspond to representative geometries in which the azurin is attached to the gold tip.

**Figure 4:**
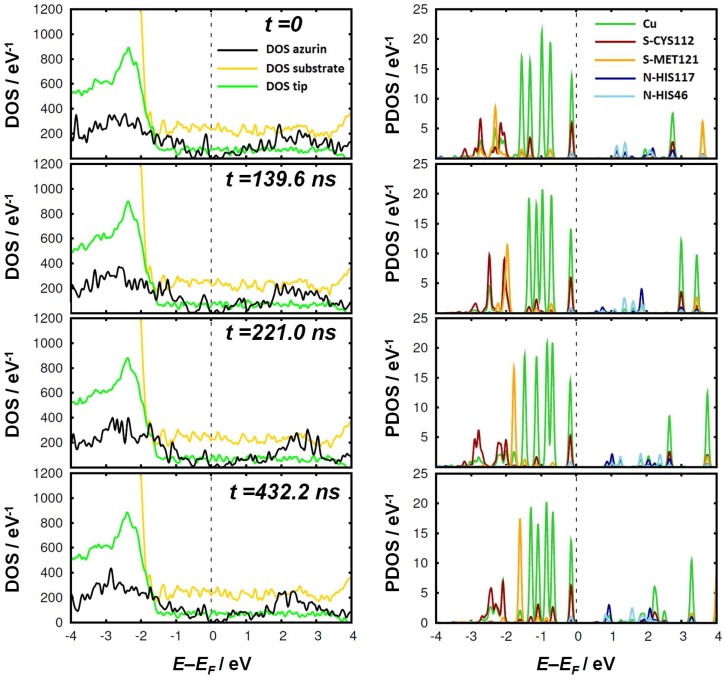
Density of states for relevant parts of the system at the four different time frames selected from the MD simulations. In the left panel, the PDOS of the azurin in the junction, the tip, and the substrate are given. In the right panel, the PDOS of some relevant atoms in the first coordination sphere of the Cu-center (green curve) are presented.

First, we focused on the density of states (DOS) of the protein within the junction together with the contribution of the tip and the substrate, shown in the left-hand panel of Figure 4. No relevant changes are observed in the contributions of the tip and the substrate, which remain unaltered during the whole process. This was expected due to their large size, which results in the typical DOS profiles of metallic surfaces. In the DOS of the azurin contribution we do not observe significant changes between the different snapshots either. However, this does not allow us to discard the possibility of more significant variations localized on specific regions of the protein, as we will show later.

In the right-hand panel of Figure 4, we show the projected density of states of the Cu-center and four atoms belonging to the first coordination sphere of the complex. This region has been claimed to play a major role in the electron transport through this protein [14,15]. The fifth ligand residue is omitted because it lacks orbitals near the Fermi energy. We observed that the typical level structure of the Cu-center remains unchanged, resembling the original gas-phase structure [11]. Something similar occurs for the S and N atoms responsible for the coordination bonds in the metal complex, which do not display any significant change during the attachment to the tip. The five *d* orbitals stay below the Fermi level, with the uppermost level very near to the Fermi level: this corresponds to the HOMO of the protein and contains contributions from S-CYS112 and also, to a lesser extent, from N-HIS117 and N-HIS46. The only remarkable change is the rearrangement of some inner levels of the S atoms in the first coordination sphere (from CYS112 and MET121) at $E-E_{F}∼-2$ eV, especially at t=221.0 ns and t=432.2 ns. A negligible impact of the second electrode was recently reported by Xie et al. [52] for oligophene dithiols and attributed to Fermi level pinning. In our case, it is rather caused by the extremely low coupling between the metal and the highly-localized orbitals of the protein. This is similar to what was previously observed for long thiolated phenyl-based molecules [53].

**Figure 5:**
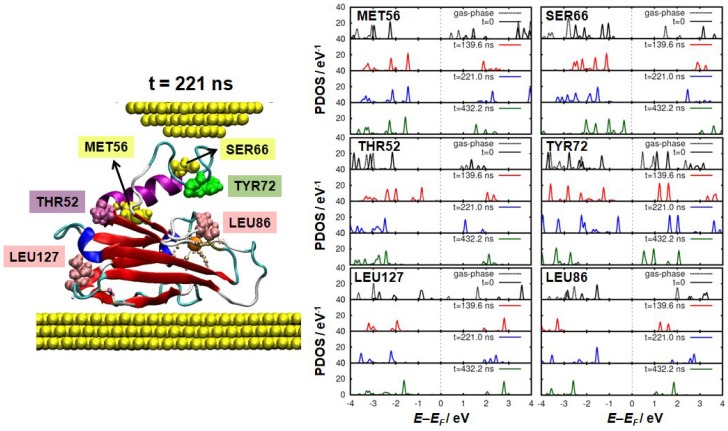
The PDOS of some key residues during the blinking event. The selected residues are highlighted in the ribbon representation of the left. The PDOS of each residue has been calculated for the initial geometry (t=0) and for the three representative frames selected of the blinking geometries. The dashed lines of the PDOS at t=0 correspond to the gas-phase calculation of the azurin.

It is hard to believe, however, that the close interaction between the tip and the protein does not lead to any variation of the electronic properties in the azurin. This prompted us to investigate the system even further. As we pointed out earlier, tracking the evolution of the electronic properties in such a large molecule (∼2000 atoms) is not a trivial task. Noticeable changes could take place in the electronic properties of some specific regions of the protein without leading to an observable net change in the total DOS due to their high localization. To obtain a further insight in this regard, we analyzed the PDOS of the 128 residues that constitute the azurin chain for the four frames obtained from the MD simulation and indicated in Figure 3e. This analysis confirmed our expectations: although the electronic density of states of the majority of the residues remains rather unaltered, there are a number of residues in which remarkable differences are observed. In the left-hand panel of Figure 5, we show the position of six of these residues within the azurin structure. The corresponding PDOS for the four frames is shown in the right-hand panel of the same figure. These residues have been selected as they are representative of more residues whose PDOS change in time in a very similar way.

For the initial geometry (t=0), we also plotted the gas-phase PDOS of the amino acids. Notice that some cases display a remarkable difference between the gas-phase PDOS of the selected residues and the PDOS of the azurin when placed in the junction. For the majority of amino acids, we observe that the PDOS of the azurin in the initial geometry is shifted to higher energies with respect to the Fermi level as compared to the gas phase. This result reveals that the protein is already locally influenced by the presence of the gold electrodes before the tip-protein contact is formed. We observed that, globally, charge transfer takes place from the azurin onto the metal. Interestingly, the amount of charge lost by the protein is higher in the initial geometry (2.70 *e*) and gradually decreases throughout the blinking event (being 2.06 *e*, 1.64 *e* and 1.29 *e* for the geometries b, c, and d of Figure 3, respectively).

The most affected residues are located over the whole protein. Some of them are found near the tip (SER66 and TYR72) or the substrate (LEU127), where a stronger interaction with the metal is expected. However, changes are also observed in a group of residues which are neither located close to the tip nor to the substrate but, rather, belong to the intermediate region in which the α-helix is separated from the β-barrel (as is the case, for instance, for THR52 or LEU86). Notice that, for most residues, the variations found in these residues are mainly rigid shifts of the PDOS that do not affect its profile. The observed changes are partly due do the interaction with the tip and partly due to the structural deformation induced by the tip attachment (as for LEU86).

As pointed out above, the changes observed in the electronic structure of the aforementioned residues do not affect either the general shape of the total DOS or the PDOS of the Cu-centered complex. Hence, one can foresee that the mechanical deformation undergone by the azurin protein (after anchoring to the tip, as in panel b–d of Figure 3) should not affect the electrical conductance significantly, either. This makes this protein an ideal candidate to be incorporated in solid-state devices. Indeed, the possibility of defining a clear conductance is a clear advantage as compared to many other systems studied in molecular electronics for which mechanical deformation and changes in the molecule-metal contact are known to induce non-negligible changes in the conductance [26,27,28]. For instance, previous studies [54,55] showed that stretching junctions based on small organic molecules can affect their electrical conductance by modifying the coupling. However, it would be difficult to observe this effect in azurin junctions because the majority of the protein orbitals are highly localized and decoupled from the metal leads. Moreover, any change of this kind in individual residues would be hidden by the overall electronic structure. Nevertheless, this should be confirmed by electron-transport calculations, a challenge that we shall face in future work.

## 4. Conclusions

We studied the mechanical deformation induced in a blue-copper azurin during the formation of a single-protein junction in an STM gap. We found that the α helix becomes separated by the β-barrel during the process. The corresponding changes in the electronic structure of the protein are, nevertheless, only localized on specific residues and do not affect the overall electronic properties of the whole system. These observations make this protein an appealing candidate to be incorporated in solid-state devices.

## Abbreviations

The following abbreviations are used in this manuscript:

STM	Scanning Tunneling Microscopy
MD	Molecular Dynamics
DFT	Density Functional Theory
